# Detection of microsatellite instability with Idylla MSI assay in colorectal and endometrial cancer

**DOI:** 10.1007/s00428-021-03082-w

**Published:** 2021-03-23

**Authors:** Iiris Ukkola, Pirjo Nummela, Annukka Pasanen, Mia Kero, Anna Lepistö, Soili Kytölä, Ralf Bützow, Ari Ristimäki

**Affiliations:** 1 Department of Pathology, HUSLAB, HUS Diagnostic Center, Helsinki University Hospital and University of Helsinki, P.O. Box 400, HUS, FI-00029 Helsinki, Finland; 2 Applied Tumor Genomics Research Program, Research Programs Unit, University of Helsinki, Helsinki, Finland; 3 Department of Gastrointestinal Surgery, Helsinki University Hospital, Helsinki, Finland; 4 Department of Genetics, HUSLAB, HUS Diagnostic Center, Helsinki University Hospital, Helsinki, Finland

**Keywords:** Colorectal cancer, Endometrial cancer, Idylla, Immunohistochemistry, Microsatellite instability, Mismatch repair

## Abstract

Universal testing of microsatellite instability (MSI) is recommended for colorectal cancer (CRC) and endometrial cancer (EC) to screen for Lynch syndrome and to aid in assessing prognosis and optimal treatment. We compared the performance of Idylla MSI test to immunohistochemistry (IHC) of mismatch repair (MMR) proteins in consecutive series of 100 CRC and 108 EC samples, as well as in retrospective series of 28 CRC and 33 EC specimens with known deficient MMR protein expression. The concordance between the Idylla test and IHC was 100% in all CRC samples (*n*=128) but lower in EC samples (87.2%; *n*=141). In the EC samples, sensitivity of Idylla test was 72.7% and specificity 100%. EC MSI/dMMR agreement was 85.4% for MLH1, 87.5% for MSH2, and only 35.3% for MSH6. When we analyzed 14 EC samples that were discrepant, i.e., dMMR using IHC and microsatellite stable using Idylla, with microsatellite markers BAT25 and BAT26, we found four cases to be replication error (RER) positive. All RER positive cases were deficient for MSH6 protein expression. We also re-analyzed EC samples with variable tumor cellularity to determine the limit of detection of the Idylla test and found that a 30% or higher tumor cellularity is required. We conclude that Idylla MSI test offers a sensitive and specific method for CRC diagnostics but is less sensitive in EC samples especially in the case of MSH6 deficiency.

**Supplementary Information:**

The online version contains supplementary material available at 10.1007/s00428-021-03082-w.

## Introduction

Microsatellites are short repetitive DNA sequences that are prone to replication errors (RER). Microsatellite instability (MSI) is caused by deficient mismatch repair (dMMR) system, leading to hypermutation phenomenon and cancer susceptibility [1, 2]. Approximately 15% of colorectal cancers (CRC) and 30% of endometrial cancers (EC) arise from MSI pathway [3]. Most MSI cancers are sporadic and account for approximately 90% of the MSI EC cases and 80% of the MSI CRC cases, which most often develops through acquired *MLH1* promoter hypermethylation [4, 5]. Lynch syndrome (LS), on the other hand, is a hereditary form of MSI most often caused by germline mutation in one of the MMR genes (*MLH1*, *MSH2*, *MSH6*, or *PMS2*) [2]. To this end, universal testing of MSI is recommended for CRC and EC patients to screen for LS and to aid in assessing prognosis and determining optimal treatment and follow-up [6–8].

Immunohistochemistry (IHC) technique is used in many pathology laboratories as a standard method to detect the loss of MMR protein expression to screen dMMR cases. The other standard method is PCR-based microsatellite test that consists of variable number and kinds of microsatellite markers, including at least mononucleotide markers BAT25 and BAT26, and is an alternative method for IHC MMR testing, especially in case of doubtful IHC results. [6] The sensitivity to detect MSI in dMMR CRC tumors using PCR-based microsatellite tests has been reported to be 89% for MLH1/MSH2 deficient but only 77% for MSH6 deficient cases [9], and in EC, the sensitivity has been estimated to range from 41 to 100% and the specificity from 69 to 89% [10].

The requirement of extensive hands-on time and trained personnel for above-mentioned IHC and PCR analysis, along with increasing demand of MSI testing, makes fast and automated molecular methods attractive alternatives. One novel way to assess the MSI status is Idylla MSI test, which analyzes a panel of seven monomorphic microsatellite biomarkers (*ACVR2A*, *BTBD7*, *DIDO1*, *MRE11*, *RYR3*, *SEC31A*, and *SULF2*) using fluorescent-labeled molecular beacons combined with PCR amplification. Idylla test is an automatic system performing all the necessary steps from a formalin-fixed paraffin-embedded (FFPE) tissue flake to MSI status information, including DNA extraction, amplification, and data analysis in 150 min [11].

The aim of this study was to evaluate the diagnostic performance of the Idylla MSI test as compared to the routine MMR IHC in CRC and EC samples. Analysis was performed in both consecutive sample series (CRC *n*=100 and EC *n*=108) and in retrospective series (CRC *n*=28 and EC *n*=33) with known dMMR IHC result. In addition, we scrutinized the minimum tumor cellularity requirement for the Idylla MSI test to detect the MSI status in the EC samples.

## Materials and methods

### Sample selection

We analyzed a prospective and consecutive series of 100 CRC samples (CRC Set I, of which one was an appendix adenocarcinoma and one a colon descendens adenoma) from patients who underwent surgical resection at Helsinki University Hospital (HUH) between February and April 2019. These patients were routinely screened for MMR proteins MLH1, MSH2, MSH6, and PMS2 in real-life diagnostic setting using IHC and experimentally tested in blinded manner for MSI using Idylla MSI test at the Meilahti Pathology Department, Helsinki, Finland. We also collected a consecutive series of 108 EC samples (EC Set I) from patients operated at HUH between February 2018 and March 2020, which were analyzed in blinded manner using Idylla MSI test. In addition to the consecutive EC Set I, we collected a historical EC series (EC Set II; *n*=33) with known dMMR protein IHC result from patients operated at HUH between January 2007 and February 2012 and reanalyzed the samples using IHC and Idylla. A retrospective CRC Set II consisted of 28 samples with known IHC result of dMMR for MSH2, MSH6, or PMS2 and operated at HUH between October 2017 and September 2020, which had not been included to the CRC Set I. This study was approved by the Ethics Committee of the Helsinki University Central Hospital.

### Immunohistochemistry

IHC was done on all CRC and EC tissue samples to detect the loss of MMR protein expression as a golden standard test using the following antibodies: MLH1 (clone ES05, diluted 1:50; Dako/Agilent, Santa Clara, CA), MSH2 (clone G219-1129, diluted 1:400; BD Biosciences, San Jose, CA), MSH6 (clone EPR3945, diluted 1:200; Abgent, San Diego, CA), and PMS2 (clone EP51, diluted 1:50; Dako/Agilent, Santa Clara). The MSH2 and MSH6 stainings were performed with Ventana BenchMark ULTRA immunostainer (Roche, Ventana Medical Systems, Tucson, AZ, USA) utilizing OptiView DAB kit (760-700, Ventana/Roche). MLH1 and PMS2 stainings were performed with Autostainer (Agilent/Dako, Santa Clara, USA) utilizing BrightVision detection kit (DPVB110HRP, Immunologic, WellMed, Duiven, the Netherlands). The loss of one or more MMR protein was defined as a dMMR, and the expression of all four MMR proteins was defined as proficient MMR (pMMR). Negative MMR protein expression was considered valid if nuclear staining in the tumor cells was absent with positive external (normal colon mucosa) and internal control staining (stromal nonneoplastic cells).

### Macrodissection

The minimum tumor cell percentage instructed by the manufacturer for Idylla MSI test is ≥ 20% for CRC samples, and for the EC samples, we used ≥ 30% proportion of tumor cells. To increase the tumor cell percentage at or above the detection limit, macrodissection was performed for the FFPE tissue blocks of 4/100 CRC Set I samples, 71/108 EC Set I samples, 26/33 EC Set II samples, and 19/28 CRC Set II samples. After macrodissection, the tumor cell percentages were 20–90% for CRC samples and 30–90% for EC samples, as estimated from the HE slides by an experienced pathologist (AR). Manufacturer’s protocol to perform the Idylla test requires a total tissue area between 25 and 300 mm^2^ with section thickness of 10 μm. For Idylla analysis, one or two 10-μm tissue sections were cut from the FFPE tissue blocks with a Leica SM2000R microtome (Leica Microsystems GmbH, Wetzlar, Germany) using aseptic conditions.

### Idylla MSI test

FFPE tissue samples were tested using automated Idylla MSI^TM^ Test (Biocartis NV, Mechelen, Belgium) that has been CE-IVD validated for CRC samples. The tissue handling and analysis were performed according to the manufacturer’s protocol. The Idylla test result is considered valid if at least five out of the seven biomarkers (*ACVR2A*, *BTBD7*, *DIDO1*, *MRE11*, *RYR3*, *SEC31A*, and *SULF2*) are fully analyzed. The presence of at least two mutant biomarkers give rise to the judgment of MSI phenotype, whereas the presence of zero or one mutant biomarker indicates MSS phenotype.

### Determination of the limit of detection

To scrutinize the limit of detection of the Idylla MSI test, we analyzed samples with variable tumor cell percentages. For that, we selected eight representative cases from the EC set I samples, which had been dMMR using IHC and MSI by the Idylla test. We then macrodissected these samples to achieve altogether 21 separate samples with tumor cellularity varying from 10 to 70%, as estimated by AR, that were analyzed with the Idylla platform.

### Replication error status using BAT25 and BAT26 mismatch markers

Discrepant samples between IHC and Idylla test were further analyzed with RER test using BAT25 and BAT26 mononucleotide repeats, in which 10-μm FFPE tissue flakes were cut from whenever 50% or more tumor cells [12] could be retrieved and DNA was extracted from the deparaffinized flakes by using a Maxwell® CSC Blood DNA Kit (Promega Corporation, Madison, WI) according to the manufacturer’s instructions. MSI status was then assessed using the two mononucleotide-repeat markers using fluorescently labeled PCR. Detection of allelic variation in both BAT25 and BAT26 was considered as a positive result representing MSI.

### Statistical analysis

The MMR IHC was considered as the golden standard reference test against which the overall agreement (concordance), sensitivity and specificity, and the positive predictive value and negative predictive value were calculated. To quantify the degree of agreement between IHC and the Idylla test, two-tailed Fisher’s exact test was used (GraphPad QuickCalcs: https://www.graphpad.com/quickcalcs/contingency2/). In order to compare the mutated biomarker spectrums between MSI CRC cases and MSI EC cases detected by the Idylla test, the unpaired *t*-test was used (GraphPad QuickCalcs: https://www.graphpad.com/quickcalcs/ttest2/). *P*-value less than 0.05 was considered as statistically significant, and results are shown as means±SD.

## Results

### Comparison between Idylla MSI analysis and IHC in colorectal cancer

One hundred consecutive CRC samples (CRC Set I) were analyzed in prospective, blinded, and real-life diagnostic setting by IHC of MMR proteins (MLH1, MSH2, MSH6, and PMS2) and Idylla MSI test. Of these patients, 44 were males and 56 females with a median age of 76 years (range from 36 to 96), and the tumors localized to the right colon (*n*=54), the left colon (*n*=33), and the rectum (*n*=11). CRC tumor cell percentages estimated for the Idylla analysis varied between 20 and 90%, and none of the Idylla test results were invalid. IHC and Idylla results showed a 100% agreement and were dMMR/MSI in 32 and pMMR/MSS in 68 of the cases (Table 1; two-tailed Fisher’s exact test, *P* < 0.0001). Based on IHC results, dMMR resulted from loss of MLH1 protein (and concomitant loss of PMS2 protein) expression in 31 cases and an isolated loss of MSH6 in one case.

**Table 1:** Concordance between MMR protein IHC analysis and Idylla MSI test in the consecutive CRC Set I (*n*=100)

		Idylla	
		MSI	MSS
IHC	dMMR	32	0
	pMMR	0	68

*dMMR*, deficient MMR protein expression, *MMR* mismatch repair, *MSI* microsatellite instable, *MSS* microsatellite stable, *pMMR* proficient MMR protein expression

**Fig. 1:**
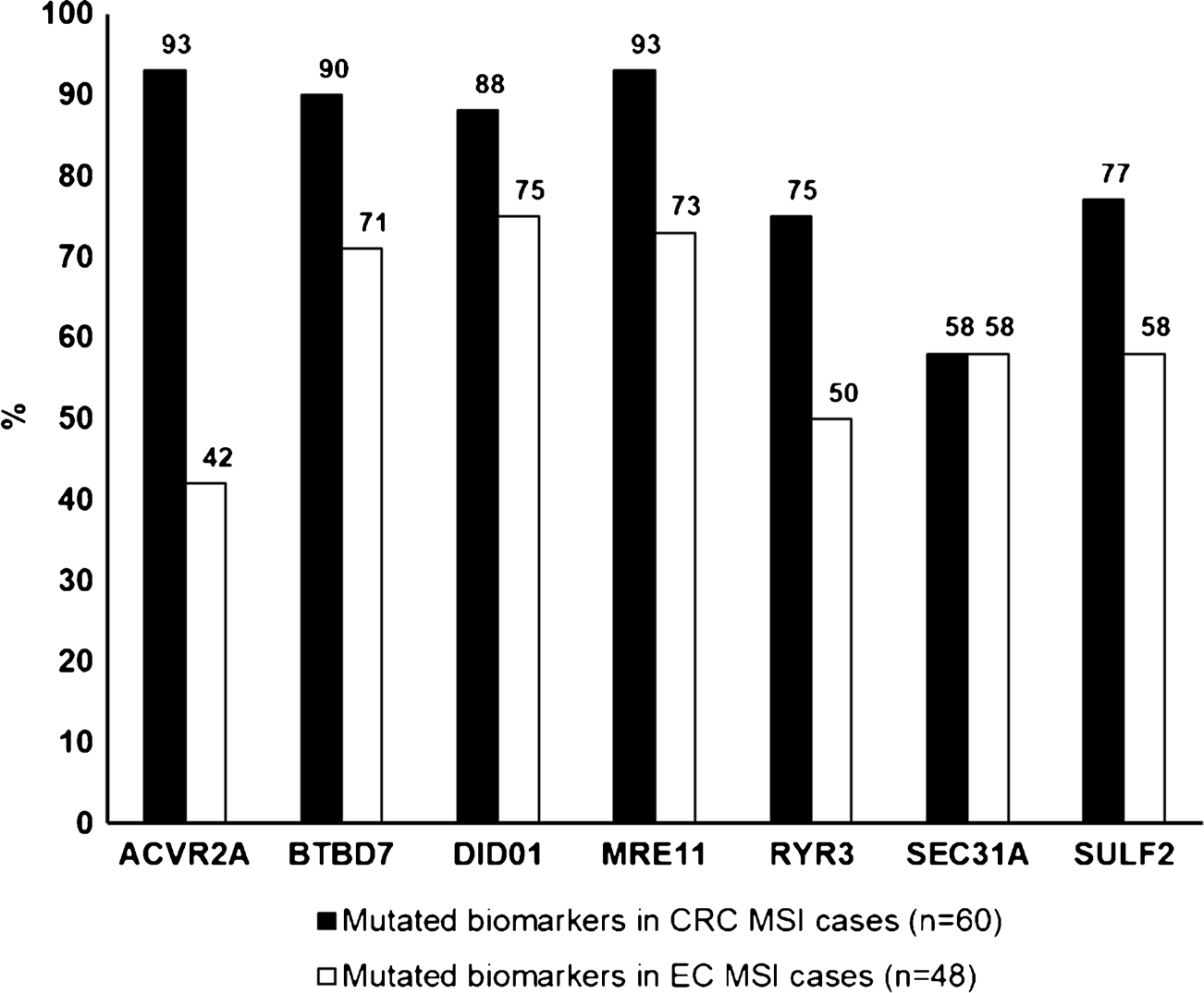
Comparison of the mutated biomarker spectrum between MSI CRC cases and MSI EC cases detected by the Idylla MSI test. Two-tailed unpaired *t*-test, *P* < 0.05

Since sensitivity of MSI detection might depend on the type of MMR gene affected, we collected 28 retrospective CRC samples (Set II) with known dMMR IHC staining pattern. Based on IHC results, there was loss of MSH2 (and concomitant loss of MSH6) protein expression in 18, an isolated loss of MSH6 protein expression in 5, and an isolated loss of PMS2 protein expression in 5. CRC tumor cell percentages estimated for the Idylla analysis varied between 20 and 80%, and none of the Idylla test results were invalid. Again, IHC and Idylla results showed a perfect 100% agreement. When the MSI cases of CRC Sets I and II were combined (*n*=60), Idylla test detected three to seven mutated biomarkers in 59/60 samples and two mutated biomarkers in one sample, whereas all the MSS samples had seven wild-type biomarkers. On average, 82.1±12.9% of the seven unstable sites were mutated in the MSI CRC cases. Two markers, *ACVR2A* and *MRE11*, were most commonly mutated (56 samples, 93.3%), and *SEC31A* was least commonly mutated (35 samples, 58.3%) (Fig. 1).

### Comparison between Idylla MSI test and IHC in consecutive series of endometrial cancer

Patients of the consecutive EC sample series (Set I, *n*=108) had median age of 71 years (range from 34 to 90). Tumor cell percentage estimated for the Idylla analysis varied between 30 and 90%. None of the Idylla test results were invalid, and 27/108 were MSI and 81/108 MSS. IHC, in turn, showed dMMR protein expression in 33/108 samples and pMMR protein expression in 75/108 samples. We thus identified 6/108 (5.6%) discrepant cases between IHC (all dMMR) and the Idylla test (all MSS), the overall agreement being 94.4% (102/108) (Table 2; two-tailed Fisher’s exact test, *P* < 0.0001). Loss of MLH1 protein (and concomitant loss of PMS2 protein) was detected in 27/33, loss of MSH2 (and concomitant loss of MSH6) protein expression in 4/33, an isolated loss of MSH6 protein expression in 1/33, and combined loss of MLH1, PMS2, and MSH6 expression in 1/33 samples. In the latter case, however, the loss of MSH6 protein expression was not homogeneous, which may suggest a somatic mutation in the microsatellites of *MSH6* gene due to the MLH1 deficiency [13]. Using IHC analysis as a reference, sensitivity of Idylla test was 81.8% and specificity 100%, while positive predictive value (PPV) was 100% and negative predictive value (NPV) 92.6%. With IHC, four of these discrepant cases were in the MLH1 deficient sample group (4/28, 14.3%), one was in the MSH2 deficient group (1/4, 25%), and one was the only MSH6 deficient sample (1/1, 100%) (Table 3).

**Table 2:** Concordance between MMR protein IHC analysis and Idylla MSI test in the consecutive EC Set I (*n*=108)

		Idylla	
		MSI	MSS
IHC	dMMR	27	6
	pMMR	0	75

*dMMR* deficient MMR protein expression, *MMR* mismatch repair, *MSI* microsatellite instable, *MSS* microsatellite stable, *pMMR* proficient MMR protein expression

**Table 3:** Agreement for each MMR protein as determined by IHC and Idylla MSI test in the consecutive EC Set I (*n*=33)

	IHC dMMR	Idylla MSI	Idylla MSS	MSI/dMMR agreement
MLH1	28	24	4	24/28 (85.7%)
MSH2	4	3	1	3/4 (75.0%)
MSH6	1	0	1	0/1 (0.0%)
Total	33	27	6	27/33 (81.8%)

*dMMR* deficient MMR protein expression, *MMR* mismatch repair, *MSI* microsatellite instable, *MSS* microsatellite stable

### Analysis of the Idylla MSI test limit of detection for endometrial cancer samples

Since there is no IVD-CE claim for the EC tissue samples, we next studied the limit of detection for the Idylla MSI test. For that, eight concordant dMMR IHC and Idylla MSI EC Set I samples were selected and macrodissected in order to obtain 21 samples with tumor cell percentage varying from 10 to 70%. The MSI result was obtained in 2/8 (25%) samples with tumor cell percentage < 20% (10–15%), in 2/3 (66.7%) samples with tumor cell percentage 25%, and in 10/10 (100%) samples with tumor cell percentage ≥ 30% (30–70%), suggesting that at least for these samples the 30% tumor cell cut off was optimal (Supplementary Table S1).

### Comparison between Idylla MSI test and IHC results in retrospective series of endometrial cancer

To further validate Idylla MSI test in EC, we collected EC Set II (*n*=33) from cases with known dMMR protein IHC diagnosis. EC patients included into this retrospective dMMR EC sample series had median age of 60 years (range from 43 to 81). First, we restained the samples with the current MMR IHC protocol to verify the dMMR status. IHC showed loss of MLH1 protein (and concomitant loss of PMS2 protein) in 13, loss of MSH2 (and concomitant loss of MSH6) protein expression in 4, and an isolated loss of MSH6 protein expression in 16. Tumor cell percentage estimated for Idylla analysis varied between 30 and 90%. The Idylla test scored all the results valid and they were MSI in 21/33 and MSS in 12/33 of the dMMR cases. We here identified 12/33 (36.3%) discrepant cases between Idylla (all MSS) and IHC (all dMMR), the overall percent agreement between the tests being 63.6% (21/33). With IHC, 10 of these discrepant cases were of the MSH6 deficient sample group (10/16, 62.5%), two were of the MLH1 deficient sample group (2/13, 15.4%), and none were of the MSH2 deficient samples (0/4) (Table 4).

**Table 4:** Agreement for each MMR protein as determined by IHC and Idylla MSI test in the EC Set II (*n*=33)

	IHC dMMR	Idylla MSI	Idylla MSS	MSI/dMMR agreement
MLH1	13	11	2	11/13 (84.6%)
MSH2	4	4	0	4/4 (100%)
MSH6	16	6	10	6/16 (37.5%)
Total	33	21	12	21/33 (63.6%)

*dMMR* deficient MMR protein expression, *MMR* mismatch repair, *MSI* microsatellite instable

**Fig. 2:**
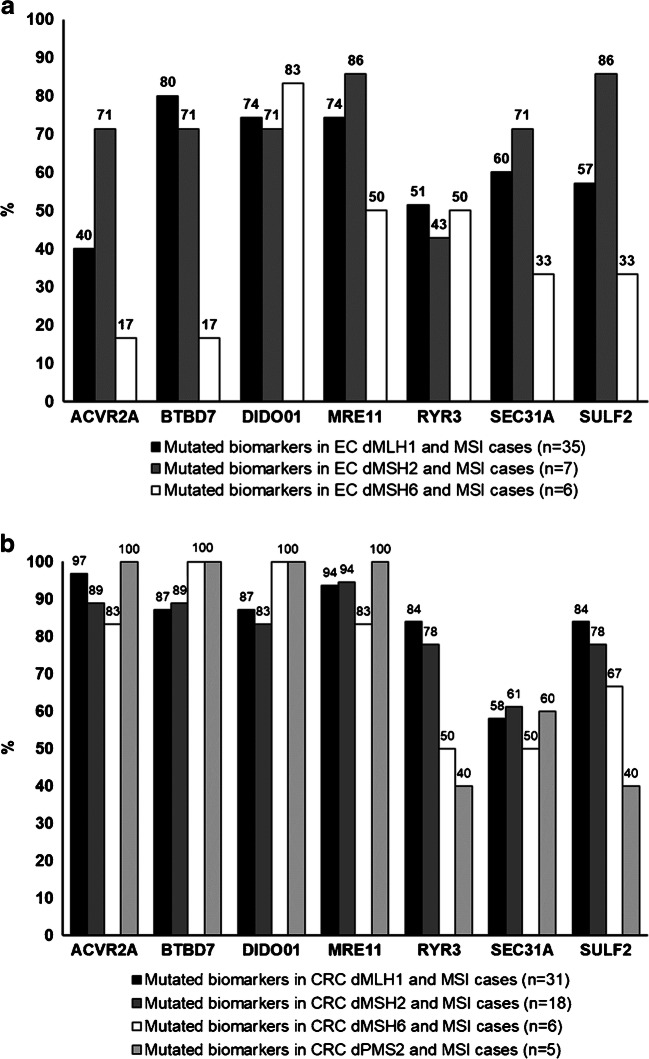
Mutated biomarker spectrums for each dMMR IHC protein in MSI EC and CRC cases detected by the Idylla MSI test. **a** Combined MSI cases from EC Sets I and II (*n*=48) and **b** combined MSI cases from CRC Sets I and II (*n*=60)

MSI/dMMR agreement for each dMMR protein group calculated from the combined EC Set I and EC Set II is presented in Table 5. In this combined EC series (*n*=48), Idylla test detected three to seven mutated biomarkers in 41/48 samples and two mutated biomarkers in 7/48 samples, whereas one MSS sample had one mutated biomarker and the rest of them had seven wild-type biomarkers. On average, 61.0±12.5% of the seven unstable sites were mutated in the MSI EC cases (Fig. 1), which was significantly lower than that of the CRC cases (82.1 ± 12.9%; *P* < 0.05). Of the mutated markers, *ACVR2A* and *RYR3* were mutated in 20 and 24 samples (41.7% and 50.0%), *SEC31A* and *SULF2* in 28 samples (58.3%), and the rest of the markers were mutated in 34 to 36 cases (70.8 to 75.0%). In combined EC Set I and Set II, mutated biomarkers detected by Idylla MSI test varied depending on the MMR protein deficiency. Especially this applied to the MSH6 deficient cases, which less frequently showed *ACVR2A* and *BTBD7* mutations (both 16.7%), but most often had mutated *DIDO01* (83.3%) (Fig. 2a). In MSH6 deficient CRC samples, similar divergences were not observed (Fig. 2b).

**Table 5:** Agreement for each MMR protein as determined by IHC and Idylla MSI test in all EC samples (EC Set I and Set II; *n*=66)

	IHC dMMR	Idylla MSI	Idylla MSS	MSI/dMMR agreement
MLH1	41	35	6	35/41 (85.4%)
MSH2	8	7	1	7/8 (87.5%)
MSH6	17	6	11	6/17 (35.3%)
Total	66	48	18	48/66 (72.7%)

*dMMR* deficient MMR protein expression, *MMR* mismatch repair, *MSI* microsatellite instable

### Analysis of the discrepant endometrial cancer cases using microsatellite markers BAT25 and BAT26

In the EC Set I, we identified six and in EC Set II 12 discrepant cases demonstrating dMMR IHC result and MSS using the Idylla test, which were first re-evaluated (tumor cell percentage) and retested with the Idylla MSI test using new paraffin slices. The result of re-testing was MSS in all 18 cases. We also re-evaluated IHC staining patterns and identified one case (B107) with heterogeneous loss of MLH1 (and concomitant PMS2) (Supplementary Fig. S1). Subclonal loss of MMR protein expression and heterogeneous dMMR IHC staining has been reported to occur in up to 7% of ECs, and exclusively in glandular endometrioid component [14], as in the case of B107. It is also noteworthy that this heterogeneous loss of MLH1 is suggestive for sporadic rather than germline deficiency. As a third method to evaluate the MSI phenotype, the discrepant cases (from EC Set I to Set II) were further tested for RER status using BAT25 and BAT26 microsatellite markers (Table 6). For this analysis, two samples (B14 and C26) did not meet the required 50% tumor cell content [12], and two (B107 and C9) did not have enough tissue available. Of the 14 samples that were subjected to RER analysis, four were shown to be positive for both BAT25 and BAT26, indicating MSI, and rest of them were negative (Table 6). These four RER positive cases had been shown to be deficient for MSH6 protein expression.

**Table 6:** Characteristics of the 18 discrepant (Idylla MSS versus IHC dMMR) EC cases

Case	Age (years)	Tumor cells (%)	Tissue area (mm^2^)	Macro-dissection	IHC (loss of MMR proteins)	Idylla MSI analysis	Number of mutated biomarkers	RER analysis (BAT25 and BAT26)
B14	69	30	300	Yes	MSH2	MSS	0/7	ND^a^
B66	79	50	40	Yes	MLH1	MSS	0/7	Negative
B79	72	50	30	Yes	MSH6	MSS	0/7	Negative
B80	73	40	50	Yes	MLH1	MSS	0/7	Negative
B97	53	60	75	Yes	MLH1	MSS	0/7	Negative
B107	77	70	100	Yes	MLH1	MSS	0/7	ND^b^
C1	59	40	50	Yes	MSH6	MSS	0/7	Negative
C4	72	60	50	Yes	MSH6	MSS	0/7	Negative
C7	58	50	300	No	MSH6	MSS	0/7	Positive
C9	66	70	15	Yes	MSH6	MSS	0/7	ND^b^
C13	63	80	75	Yes	MSH6	MSS	0/7	Negative
C17	78	80	75	Yes	MSH6	MSS	0/7	Positive
C19	69	40	100	Yes	MLH1	MSS	0/7	Negative
C20	61	80	150	No	MSH6	MSS	0/7	Negative
C21	49	80	75	Yes	MSH6	MSS	0/7	Positive
C22	72	80	100	Yes	MLH1	MSS	1/7	Negative
C26	80	30	25	Yes	MSH6	MSS	0/7	ND^a^
C29	58	60	100	Yes	MSH6	MSS	0/7	Positive

*dMMR* deficient MMR protein expression, *IHC* immunohistochemistry, *MMR* mismatch repair, *MSI* microsatellite instable, *MSS* microsatellite stable, *ND* not determined, *RER* replication error

^a^Tumor cell percentage less than 50%

^b^Not enough tissue available

## Discussion

In this study, we evaluated the diagnostic performance of Idylla MSI test in detecting MSI in CRC (*n*=128) and EC (*n*=141) samples. The Idylla test showed 100% concordance with the MMR IHC results in the CRC cohort but only 87.2% concordance in the EC cohort. None of the Idylla analysis failed due to technical errors or sample-related problems. Strength of the study includes real-life prospective blinded design of the 100 consecutive CRC cases. We were not able to perform identical study protocol for the EC cases, which were analyzed in two cohorts, i.e., a consecutive and blinded Set I and a separate historical Set II with known dMMR IHC status. As compared to IHC, sensitivity of the Idylla test was 72.7% and specificity 100% in EC samples. Eighteen discrepant EC cases between IHC and Idylla test were detected, and we were able to subject 14 of these to RER analysis using two mononucleotide markers BAT25 and BAT26. Four cases were identified that were repeatedly MSS in the Idylla analysis but were found to be MSI when using the BAT25 and BAT26 markers. All these cases were deficient for expression of MSH6 protein. Weakness of this study was that we used only two mononucleotide microsatellite markers instead of five, which may have been more sensitive in detecting MSI [6].

One explanation for discrepancies between IHC and DNA-based assay could depend on the amount of tumor cells included to the FFPE flakes. To this end, we scrutinized the minimum tumor cellularity requirement for Idylla MSI test in the EC samples. Our data suggest that at least 30% EC cell percentage is required for the Idylla analysis. Further, low tumor cell number does not explain the discrepant cases that we found between IHC and the Idylla test, since all four RER-positive (BAT25 and BAT26 positive) cases had tumor cell percentages between 50 and 80%. It should be pointed out that we also included MSH6 losses to the CRC cohort (*n*=6) that were all correctly identified as MSI using the Idylla test. Our results are supported by previous findings showing that MSI in EC demonstrates a higher frequency of minimal (1–2 nucleotide) microsatellite shifts especially in the case of MSH6 loss, which is a challenge for DNA-based MSI assays [9, 15, 16]. Importantly, detection of MSH6 deficiency is in parts crucial, since females carrying a pathological MSH6 mutation are reported to be at especially high risk of endometrial cancer compared with other LS-related cancers [17].

Previous reports have also found a very high concordance (97.6–100%; *n*=42–105) of the Idylla MSI test with reference tests in retrospective CRC samples [18–20]. In a larger sample set, Zwaenepoel et al. reported the Idylla test to show 98.7% overall agreement using historical and partially dMMR-enriched IHC data in 330 CRC samples [21]. In this report, one case was MSS in Idylla analysis, whereas IHC was dMMR and Promega MSI analysis was MSI and interestingly tumor mutation burden was very high, whereas three IHC dMMR cases showed MSS in DNA-based assays and low tumor mutation burden. In addition, a multi-center real-life global study including 44 clinical centers and 1301 CRC samples showed the concordance level between the Idylla test and IHC to be as high as 96.4%, with Idylla having lower failure rates [11].

Excellent accuracy of the Idylla MSI test in CRC samples has encouraged to study the diagnostic performance of this novel assay in other solid tumors as well. Farmkiss et al. compared retrospectively IHC and the Idylla test across 50 biopsies of gastric adenocarcinoma, scoring concordant results in 48 samples with a 95.7% sensitivity and 100% specificity [22]. Pécriaux et al. have evaluated the Idylla test in a panel of solid tumors, including 15 EC samples using extracted DNA, and reported sensitivity of 89% and specificity of 100% to detect MSI status in EC samples as compared to IHC [23]. It is important to note that all nine MSI cases in this report were MLH1 deficient. Gilson et al. reported similarly with 15 EC samples a 100% specificity and sensitivity with the Idylla test as compared to previously used PCR-based Promega MSI analysis, but if compared to IHC, the sensitivity seems to be 80–90% and specificity 100% [24]. Our study agrees with these reports on the excellent specificity of the Idylla MSI test in EC diagnostics, but the sensitivity when compared to IHC was clearly lower in our study (72.7%), which to our knowledge is the largest EC study thus far (*n*=141).

Our study showed significant differences in the mutated biomarker spectrum between CRC and EC MSI cases detected by the Idylla MSI test. First, on average 82.1% of the seven unstable sites were mutated in MSI CRC, whereas only 61.0% of the sites were mutated in MSI EC cases. This is in line with the previous publication reporting lower proportion of unstable markers per tumor in EC as compared to CRC, even in the tumors originating from the same LS patient [25]. Second, our results implicate that mutated biomarkers *ACVR2A* and *RYR3* are more frequently mutated in CRC when compared to EC, whereas *SEC31A* seems to be mutated by the same frequency in both CRC and EC MSI samples. Previously, Kim et al. have showed that *ACVR2A* is indeed a gene harboring frameshift microsatellite instability specifically in CRC and less often in EC genomes [26]. They also demonstrated *SEC31A* to be a gene with no extreme specificity to harbor frameshift MSI in either CRC or EC genomes. Further, the multicenter Idylla MSI test study of CRC reported *ACVR2A* to be the most often mutated biomarker (94.0%) and *SEC31A* to be the least mutated marker (60.9%) [11], and our frequencies are in line with a sequencing study demonstrating *ACVR2A* mutations in 92% and *SEC31A* mutations in 54% of one hundred MSI CRC cases [27].

In conclusion, our study reinforces the accuracy of the fast and automated Idylla MSI test to detect MSI phenotype in CRC samples. In EC samples, Idylla test is 100% specific, but sensitivity is compromised especially in the case of MSH6 deficient tumors. We conclude that the Idylla test offers a sensitive and specific method for CRC diagnostics, but it should be validated for each tumor type separately in a relatively large material before applying it to diagnostic use.

## Supplementary Information

ESM 1 (PDF 293 kb)

## Data Availability

The data obtained during the current study are available from the corresponding author AR on reasonable request.

## Abbreviations

CRC: Colorectal cancer
dMMR: Deficient MMR
EC: Endometrial cancer
FFPE: Formalin-fixed paraffin-embedded
HUH: Helsinki University Hospital
IHC: Immunohistochemistry
LS: Lynch syndrome
MMR: Mismatch repair
MSI: Microsatellite instability
MSS: Microsatellite stable
NGS: Next-generation sequencing
NPV: Negative predictive value
pMMR: Proficient MMR
PPV: Positive predictive value
RER: Replication error
