# Supplementary material for: Detection of microsatellite instability with Idylla MSI assay in colorectal and endometrial cancer
Source: Virchows Arch. 2021 Mar 23;479(3):471–9. doi: 10.1007/s00428-021-03082-w (PMC8448708; doi:10.1007/s00428-021-03082-w)
Supplement: Supplementary file 1 — (PDF 293 kb) [file 428_2021_3082_MOESM1_ESM.pdf]

Virchows Archiv

Supplementary Information

for

Detection of microsatellite instability with Idylla MSI assay in colorectal and endometrial cancer

Iiris Ukkola[1,2], Pirjo Nummela[2], Annukka Pasanen[1], Mia Kero[1], Anna Lepistö[2,3], Soili Kytölä[4], Ralf Bützow[1,2] & Ari Ristimäki[1,2]

E-mail of corresponding author: ari. ristimaki (at) helsinki. Fi

[1]Department of Pathology, HUSLAB, HUS Diagnostic Center, Helsinki University Hospital and University of Helsinki, Helsinki, Finland

[2]Applied Tumor Genomics Research Program, Research Programs Unit, University of Helsinki, Helsinki, Finland

[3]Department of Gastrointestinal Surgery, Helsinki University Hospital, Helsinki, Finland

[4]Department of Genetics, HUSLAB, HUS Diagnostic Center, Helsinki University Hospital, Helsinki, Finland

Supplementary Table S1 Characteristics of the performance of Idylla MSI test in respect of variable tumor cell percentage

			Biomarker								
**Case**	**Tumor cells (%)**	**Tissue area (mm^2)**	***ACVR2A***	***BTBD7***	***DIDO1***	***MRE11***	***RYR3***	***SEC31A***	***SULF2***	**Number of mutated biomarkers**	**Idylla MSI analysis**
B04	15	150	-	-	-	-	-	-	-	0/7	MSS
	30	300	-	-	+	+	-	-	-	2/7	MSI
	60	50	-	-	+	+	-	-	-	2/7	MSI
B05	10	150	-	-	-	-	-	-	-	0/7	MSS
	30	140	-	+	-	-	-	-	+	2/7	MSI
	70	80	-	+	+	+	-	+	+	5/7	MSI
B09	15	300	-	-	-	-	-	-	-	0/7	MSS
	30	25	-	+	+	+	+	+	+	6/7	MSI
B10	15	270	-	-	-	-	-	-	-	0/7	MSS
	50	270	+	+	+	+	+	-	+	6/7	MSI
B35	15	150	-	-	-	-	-	-	-	0/7	MSI
	40	60	+	-	-	+	-	-	+	3/7	MSI
B37	15	300	-	+	+	-	+	-	-	3/7	MSI
	25	100	-	-	+	-	+	-	-	2/7	MSI
	60	25	-	+	+	-	+	+	+	5/7	MSI
B38	15	300	-	-	-	-	-	-	-	0/7	MSS
	25	150	-	-	-	-	-	-	-	0/7	MSS
	50	50	-	-	+	+	-	-	+	3/7	MSI
B54	15	300	-	+	+	+	+	+	+	6/7	MSI
	25	150	-	+	+	+	+	+	+	6/7	MSI
	60	55	-	+	+	+	+	+	+	6/7	MSI

MSI, microsatellite instable; MSS, microsatellite stable

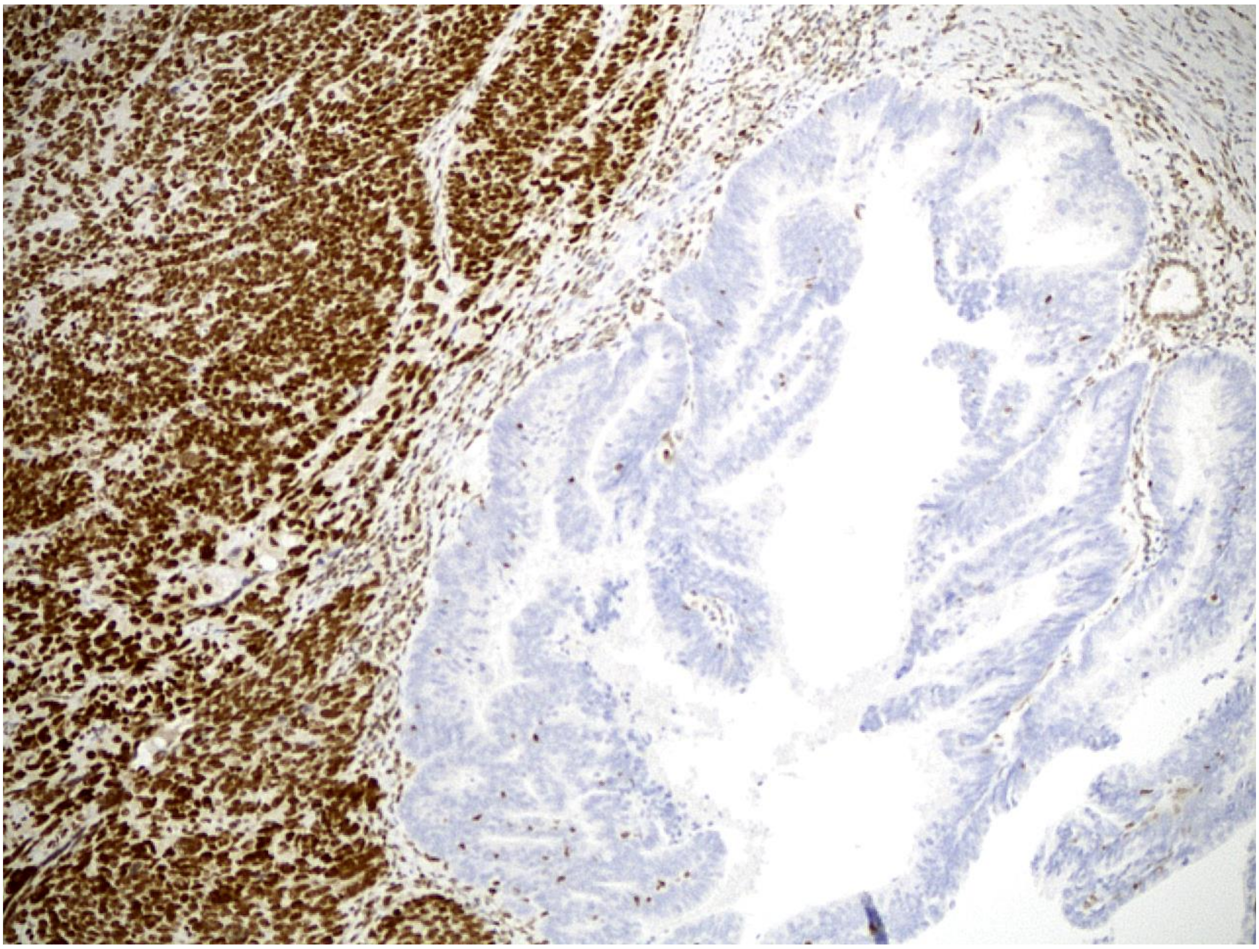

Supplementary Fig. S1 Endometrial cancer with heterogeneous loss of MLH1 (case B107). On left is shown solid component with intact MLH1 expression, whereas on the right is glandular endometrioid component with loss of MLH1 expression in cancer cells and intact MLH1 expression in intraepithelial lymphocytes (a concomitant loss of PMS2 protein expression was also detected in cancer cells, whereas MSH2 and MSH6 protein expression was intact, not shown). Original magnification 200x, DAB was used as a chromogen.
